# Muscle metastases from gallbladder cancer

**DOI:** 10.3332/ecancer.2011.236

**Published:** 2011-11-17

**Authors:** L Gilardi, G Paganelli

**Affiliations:** Division of Nuclear Medicine, European Institute of Oncology, via Ripamonti 435, 20141 Milano, Italy

## Abstract

Gallbladder cancer is a highly fatal disease with poor prognosis; indeed, a high proportion of tumours are diagnosed at an advanced stage. Metastases are commonly detected in regional lymph nodes, liver and peritoneum, but unusual sites of spread such as thyroid and breast have also been described in the literature.We report a case of unsuspected muscle metastases, associated to pulmonary metastases, detected by FDG-PET/CT in a patient with previously removed gallbladder cancer and persistent episodes of haemoptysis.

## Introduction

Gallbladder cancer is the most common malignancy of the biliary tract and is a highly fatal disease with poor prognosis. This is due to the anatomical position of the gallbladder and to the high proportion of advanced tumours at the time of presentation.

The modalities of dissemination are direct, lymphatic, vascular, neural, intraperitoneal and intraductal. After regional lymph nodes, the most common sites of distant metastases are the peritoneum and the liver. Occasionally, spreads to the lung and pleura are also reported [1].

Unusual sites of spread such as thyroid, subcutaneous tissue, breast, heart and skin have also been described in the literature [2–5].

We report a case of unsuspected muscle metastases, associated to pulmonary metastases, detected by FDG-PET/CT in a patient with previously removed gallbladder cancer and persistent episodes of haemoptysis.

## Case report

In March 2010, a 76-year-old man arrived at our department to undergo an FDG-PET/CT after persistent episodes of haemoptysis.

Previously the patient underwent radiotherapy for a laryngeal squamous cell carcinoma (cT2N0) (1998) and a central hepatectomy for an adenocarcinoma of the gallbladder (pT4N0) (July 2009). The gallbladder tumour was preoperatively staged by a PET/CT scan and by a cholangio-magnetic resonance. The first one showed only pathological FDG uptake in the primary tumour, while the second highlighted the involvement of the fourth and the fifth hepatic segment.

No treatments were performed after surgery and no residual disease was detected at the contrast-enhanced abdominal CT in November 2009. However, a small nodule deserving close follow-up was described at the base of the right lung.

**Figure 1:**
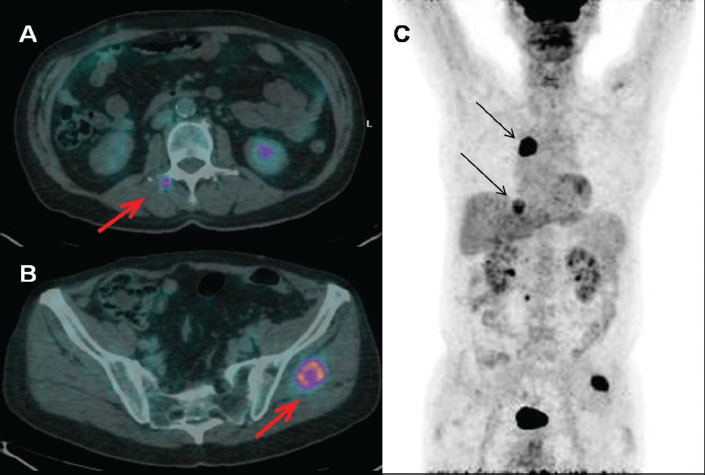
Increased uptake of the radiopharmaceutical in muscle and pulmonary metastases (FDG-PET/CT). A: right para-vertebral muscle close to the soma of L2 (fused transaxial images: red arrow); B: left gluteus muscle (fused transaxial images: red arrow); C: pulmonary lesions at right upper lobe and at the ipsilateral base (maximum intensity projection image: black arrows).

In March 2010, we performed a PET scan, using a PET/CT scanner (Discovery ST; GE Medical Systems, Waukesha, WI). A whole-body scan, starting 45 min after i.v. injection of 295 MBq of 18F-FDG, was acquired from head basis to pelvis, with patients in the supine position with arms extended above the head. Serum glucose level, determined before injection, was 104 mg/dl (the patient had no history of diabetes). The scan showed pathological radiotracer uptake in two lung nodules localized in the right upper lobe and in the ipsilateral base; the latter was already described at the CT scan in November 2009. Unsuspected lesions in the left medium gluteus muscle and in the right para-spinal muscle close to the body of L2 were also detected (Figure 1).

The patient underwent a fine needle aspiration biopsy of the gluteal lesion. A localization of poorly differentiated carcinoma consistent with gallbladder origin was found and surgery was performed. Radiation treatment was carried out on the para-spinal muscle injury (30 Gy in three fractions) and a systemic therapy with gemcitabine was set up.

In July 2010, a restaging CT showed a substantial stability of pulmonary lesions but a progression of disease in muscle (new para-spinal lesion in thorax and an increase in the size of the right para-spinal lumbar injury), lymph node, adrenal gland and bone (appearance of para-tracheal adenopathy, left adrenal nodule and lesion at the anterior tract of the second left rib).

A second-line chemotherapy with oxaliplatin and fluorouracil was then started and a radiation treatment (15 Gy in three fractions) on the thoracic para-spinal muscle lesion was also applied.

Unfortunately, in September 2010, a further restaging CT showed disease progression with an increase in size of nearly all metastatic sites.

Radiation treatments with stereotactic technique were performed with palliative intent on the lung nodule at right lower lobe and on the rib lesion.

After all these treatments, in October 2010, the patient was in good general condition, he had a Karnofsky Performance Status of 90 and controlled thoracic and right lumbar pain through analgesic therapy.

A new session of radiotherapy was planned to treat the disease in the left adrenal gland but the patient was hospitalized for post-traumatic brain haemorrhage and died ten days later.

## Discussion

FDG-PET/CT is taking on an essential role in the evaluation of many types of cancer, both in staging and in follow-up [6].

It is also becoming an important tool in evaluating response to therapy, field where new assessment criteria have been proposed, and in defining the target volume for radiotherapy planning [7,8].

The role of FDG-PET/CT in gallbladder cancer is yet ill-defined and some studies have tried to clarify this issue.

Corvera *et al* reported high sensitivity of PET for the detection of primary tumours, suggesting an exploratory surgery in the case of FDG uptake restricted to the gallbladder only if other imaging studies confirm that the tumour is resectable. Indeed PET does not provide sufficient anatomical detail about local resectability but it has an important role in detecting occult metastatic disease, leading to a stage migration and changing the treatment in a significant number of patients [9]. Moreover, Petrowsky *et al* detected each gallbladder cancer in their study population, regardless of the primary or recurrent nature of the tumour, showing a sensitivity of 100% (specificity, positive predictive value, negative predictive value and accuracy were not calculated since only one patient with benign disease suspicious for gallbladder cancer was imaged). However, this result should be carefully evaluated, given the limited number of patients with gallbladder cancer in this study (only 14 of 61). The authors also confirmed the role of PET in the detection of occult metastases from gallbladder cancer not diagnosed by conventional imaging, underlining its usefulness in the selection of patients who should be precluded from surgical resection [10]. This is also true in assessing the extent of disease in incidental gallbladder cancer, that is an unsuspected invasive carcinoma identified in the final pathology report after cholecystectomy [11]. Furthermore, PET/CT has also proven to be superior to multidetector row CT (MDCT) in the evaluation of distant site of disease in patients with gallbladder carcinoma and cholangiocarcinoma. Lee *et al* detected distant metastases in 19 of 82 patients (six of 16 with gallbladder cancer, nine of 17 with intrahepatic cholangiocarcinoma and four of 49 with extrahepatic cholangiocarcinoma). PET/CT and MDCT showed a sensitivity of 94.7% (18/19) and 63.2% (12/19), respectively (*P* = 0.02 [12]). Even in this study the small number of patients with gallbladder cancer should be taken into account as a factor affecting the conclusions.

As regards the detection of metastatic lymph nodes, contrasting results can be found in the literature. Petrowsky *et al* detected disease in only two of 17 patients with gallbladder cancer and cholangiocarcinoma, with a sensitivity of 12% [10]. Whilst the detection rate of regional lymph node metastases by PET/CT was 82.1% in Lee’s study. This result could be partially attributed to the advanced AJCC stage of the evaluated patients [12].

The diagnostic accuracy of PET may be limited by the extent of the disease, namely small-volume peritoneal disease that is below the limit of detection. Additionally, problems related to coexisting inflammation, resulting in increased glucose metabolism, could confound the diagnosis in some patients. This limitation is important to recognize, because many patients with gallbladder cancer are diagnosed after undergoing cholecystectomy for benign disease and often have varying degrees of postoperative inflammation associated with normal healing [9].

Not much is known about the usefulness of PET in the follow-up of patients with gallbladder cancer, probably due to the high mortality rate of these tumours, with a 5-year overall survival less than 5%.

Indeed, in our clinical practice, we do not frequently perform a PET/CT scan to patients with gallbladder cancer. Even in the patient that we described, the exam was carried out for the presence of a sign not directly related to this cancer. The persistent episodes of haemoptysis could indeed have been related to a recurrence of the already treated laryngeal carcinoma. However, the discovery of lung metastases, infrequent but occasionally observed in gallbladder carcinoma, and of muscle metastases, never reported in literature previously, led to a multidisciplinary approach to the patient, who benefit from it till his accidental death.

Gallbladder cancer has a remarkable propensity to spread early by direct extension into the liver and other adjacent organs. The gallbladder has a thin wall, a narrow lamina propria, and only a single muscle layer. Therefore, tumour invades into the liver at a thickness at which in other organs it would be encountering a second muscle layer. Once it penetrates the thin muscle layer, it has access to major lymphatic and vascular channels. Haematogenous metastases tends to be from invasion into small veins extending directly from the gallbladder into the portal venous system, leading to hepatic metastases in segments IV and V. The only common extra-abdominal site of metastases is the lung [13].

Subsequent systemic spread is unpredictable and it could involve several organs, as reported both previously in the literature mentioned in our article and in our case report.

## Conclusions

In conclusion, the early detection of metastases, mostly in unknown or unpredictable sites, may lead to an improvement of the subsequent treatments, with a multidisciplinary approach aiming to obtain the best quality of life for the patient.

Obviously, these are only impressions, and dedicated prospective studies are needed to define the role of PET/CT in the postoperative follow-up of gallbladder carcinoma.
